# Factors related to mouth breathing syndrome in preschool children and the effects of incompetent lip seal: An exploratory study

**DOI:** 10.1002/cre2.661

**Published:** 2022-09-15

**Authors:** Emi Inada, Issei Saitoh, Yasutaka Kaihara, Daisuke Murakami, Yukiko Nogami, Yuki Kiyokawa, Reira Tanaka, Kensuke Sakata, Youichi Yamasaki

**Affiliations:** ^1^ Department of Pediatric Dentistry Kagoshima University Graduate School of Medical and Dental Sciences Kagoshima Japan; ^2^ Department of Pediatric Dentistry Asahi University School of Dentistry Gifu Japan; ^3^ Dental Hygiene Course Ogaki Women's College Gifu Ogaki‐shi Japan; ^4^ Division of Pediatric Dentistry, Graduate School of Medical and Dental Science Niigata University Niigata Chuo‐ku Japan

**Keywords:** factor analysis, incompetent lip seal, mouth breathing syndrome, nasal disease

## Abstract

**Objectives:**

A set of orofacial signs and symptoms completely or partially present in individuals who replace the correct pattern of nasal breathing with an oral or mixed pattern is defined as mouth breathing syndrome (MBS). In a previous report, it was clarified that an incompetent lip seal (ILS) affected the occurrence of MBS among primary school children. However, the factors related to MBS and the effect of ILS in preschool children remain unclear. The purpose of this study was to clarify the factors relevant to MBS in preschool children and investigate the relationship of ILS to MBS.

**Material and Methods:**

We surveyed 285 preschool children between 3 and 5 years of age. Their guardians completed the questionnaire, which consisted of 44 questions regarding the children's daily health conditions and lifestyle habits. To classify the closely related questions into their respective factors and to examine the strength of the correlation between the newly revealed factors, an exploratory factor analysis with promax rotation was performed.

**Results:**

The factor analysis identified nine items representing four factors. Factors 1–4 were defined as “diseases of the nose,” “ILS,” “problem with swallowing and chewing,” and “eating and drinking habits,” respectively. Factor 2 most strongly correlated with Factor 1, and both Factors showed a relatively strong correlation with Factor 3.

**Conclusions:**

The initial stage of MBS may be present in preschool children. ILS and diseases of the nose can cause poor development of oral functions, such as breathing and eating.

## 1 INTRODUCTION

Mouth breathing can cause malalignment of teeth, facial deformities, oral and dental disorders, poor overall growth, and worsening of the symptoms of other illnesses (Campanha et al., 2010; Choi et al., 2016; Okuro et al., 2011). A set of signs and symptoms completely or partially present in individuals who, for various reasons, replace the correct pattern of nasal breathing with an oral or mixed pattern, is defined as mouth breathing syndrome (MBS) (Conti et al., 2011). In the past, we studied MBS and its related factors among primary school children. We reported that an incompetent lip seal (ILS) was representative of the physical appearance of mouth breathers and that it affects diseases of the nose and throat, oral diseases, such as caries and gingivitis, and oral functions, such as eating and drinking (Saitoh et al., 2018).

A major cause of an ILS is mouth breathing due to nasal obstruction or deformity (Kotecha, 2011; Sabashi et al., 2011). The other causes include difficulty in closing the lips due to an imbalance of the anterior teeth arch and maxillofacial morphology and dysfunction of the perioral muscles (Jung et al., 2010; Mizuno et al., 2014). In Japan, Nogami et al. conducted a national, large‐scale epidemiological study to reveal the prevalence of ILS in children. They surveyed 3399 children between 3 and 12 years of age using a questionnaire regarding daily health conditions and lifestyle habits and concluded that 30.7% of the participants exhibited an ILS and that its prevalence increased with age. Further, they revealed that the orofacial morphology, mouth breathing, and possibly, allergic rhinitis were correlated with ILS (Nogami et al., 2021).

The position of the teeth and the dental arch form are stabilized by the balance of forces applied by perioral tissues, such as the lips, cheeks, and tongue to the teeth during rest or activities like eating, chewing, and speaking (Hsu & Yamaguchi, 2012; Nagaiwa et al., 2016; Nishiura et al., 2015; Sabashi et al., 2011; Weinstein et al., 1963). It has long been advocated as the “equilibrium theory” by Brodie (1954) and Moyers (1972). If this balance is lost, the dental arch form would collapse due to the constant separation of the upper and lower lips (Harari et al., 2010; Lofstrand‐Tidestrom et al., 1999). To examine the influence of an ILS on the form of facial soft tissues in preschool children 3–5 years of age, we had previously measured the differences in the three‐dimensional facial images between children with and without ILS (Inada et al., 2021). We observed that the influence of an ILS on the facial form begins to appear even before 3 years of age, and children with ILS have anteriorly prominent subnasales and lips and a flatter nose.

The above‐described findings helped clarify that the prevalence of ILS between 3 and 12 years of age is not low, ILS affects the occurrence of MBS among primary school children, and the influence of an ILS on the facial form becomes apparent in preschool children (Inada et al., 2021; Nogami et al., 2021; Saitoh et al., 2018). However, the effect of ILS and factors related to MBS in preschool children have not yet been elucidated. The early improvement of mouth breathing in children is expected to contribute to the maintenance/enhancement of their health and quality of life. Therefore, clarifying the factors related to MBS in growing children can improve its diagnosis and management.

The purpose of this study was to clarify the factors related to MBS in preschool children and investigate the relationship of ILS to MBS.

## 2 MATERIALS AND METHODS

### 2.1 Human subjects and questionnaire

We surveyed 285 preschool children (150 boys and 135 girls) between 3 and 5 years of age. None of the participants had extensive dental caries extending to the pulp or crown destruction, lip dysfunction, malocclusion, or mandibular dysfunction. We asked the legal guardians of the children to fill out a questionnaire. In this study, we adopted the same questionnaire used by Nogami et al. (2021) in a previous study on the prevalence of ILS during growth periods throughout Japan. The questionnaire comprised 44 questions regarding the child's daily health condition and lifestyle habits that might be linked to MBS (Table 1). The responses were provided on a two‐point scale (yes or no). This study was approved by the Epidemiological Ethics Committee of Kagoshima University Graduate School of Medical and Dental Sciences (No. 378), and informed consent was obtained from the subjects' parents or guardians before their enrollment.

**Table 1:** Questionnaire on conditions that might be linked to MBS

Question item	
Q1 Do you get tired easily?	Q23 Do you have an anterior open bite?
Q2 Are you good riser?	Q24 Can you talk clearly?
Q3 Are you good at exercising?	Q25 Are your lips often chapped?
Q4 Are you a restless sleeper?	Q26 Are your lips thick?
Q5 Do you have round shoulders?	Q27 Is your upper lip turned upward?
Q6 Does your nose become stuffed easily during the day?	Q28 Are your teeth visible between your upper and lower lips?
Q7 Does your nose become stuffed easily while sleeping?	Q29 Are your lips droopy?
Q8 Do you sneeze often?	Q30 Are your lips often cracked?
Q9 Do you often have a runny nose?	Q31 Are your gums often swollen?
Q10 Do you often have a nosebleed?	Q32 Are your gums easily stained?
Q11 Do you often have a sore throat?	Q33 Are your teeth easily stained?
Q12 Do you have swollen tonsils?	Q34 Do you often have canker sores?
Q13 Do you often fail to listen?	Q35 Do you have tartar build‐up?
Q14 Are you a habitual snorer?	Q36 Do your meals consist of small servings?
Q15 Is your mouth often dry?	Q37 Do you prefer soft food?
Q16 Do people tell you that you have bad breath in the morning?	Q38 Do you drink water during meals?
Q17 Do people tell you that you have bad breath during day?	Q39 Do you eat fast?
Q18 Is your mouth often open during the day?	Q40 Are you a picky eater?
Q19 Do you sleep with your mouth open?	Q41 Do you chew food well?
Q20 Can you keep your mouth closed for about 1 min?	Q42 Are you a noisy eater?
Q21 Do you have an over bite?	Q43 Do you keep your mouth closed when you eat?
Q22 Do you have an under bite?	Q44 Do you have food left in your mouth for a long time?

Abbreviation: MBS, mouth breathing syndrome.

### 2.2 Statistical analysis

When there is a large number of questions in a questionnaire, answers to several questions may strongly correlate. Therefore, multiple highly correlated questions were divided into groups so that the common factors between them would become apparent. To (1) classify the closely related questions into their respective factors and (2) examine the strength of the correlations between the newly revealed factors, we performed an exploratory factor analysis (maximum‐likelihood method and promax rotation).

The number of factors was determined based on the Kaiser‐Guttman rule (eigenvalue of 1 or more) and a scree plot. Statistical analyses were performed using IBM SPSS Statistics for Windows (version 20; SPSS Inc.), and statistical significance was set at *p* < .05.

## 3 RESULTS

As a result of the exploratory factor analysis, 9 out of the 44 questions were selected as items related to MBS and classified into four factors (Table 2). The Kaiser–Meyer–Olkin measure was 0.608 and Bartlett's test of sphericity yielded *p* < .001, showing the validity of the factor analysis. The cumulative contribution ratio was 44.87%.

**Table 2:** Pattern matrix of the factor analysis

Item no.	Question item	Factor 1	Factor 2	Factor 3	Factor 4
(Q‐6)	Does your nose become stuffed easily during the day?	1.044	−0.147	−0.017	0.077
(Q‐7)	Does your nose become stuffed easily while sleeping?	0.491	0.365	−0.006	−0.147
(Q‐18)	Is your mouth often open during the day?	0.022	0.614	0.047	−0.006
(Q‐29)	Are your lips droopy?	−0.101	0.522	−0.196	0.052
(Q‐19)	Do you sleep with your mouth open?	0.019	0.470	0.177	0.114
(Q‐43)	Do you keep your mouth closed when you eat?	0.042	0.049	−0.738	−0.001
(Q‐42)	Are you a noisy eater?	0.013	0.001	0.605	−0.017
(Q‐40)	Are you a picky eater?	−0.027	0.014	−0.003	0.634
(Q‐37)	Do you prefer soft food?	0.066	0.098	−0.015	0.474

We defined Factor 1 as “disease of the nose,” based on the question “Does your nose become stuffed easily (during the day, while sleeping)?” Factor 2 was defined as “ILS” based on the factors loading of “Is your mouth often open during the day?,” “Are your lips droopy?,” and “Do you sleep with your mouth open?” Similarly, based on the factor loading and content of the other questions, Factors 3 and 4 were defined as “problems with swallowing and chewing” and “eating and drinking habits,” respectively.

Table 3 shows the factor correlation matrix. Factor 2 was most strongly correlated with Factor 1. Factor 3 showed a relatively strong correlation with Factors 1 and 2. And Factor 4 showed moderate correlations with the other factors.

**Table 3:** Factor correlation matrix

			Factor 3	Factor 4
	Factor 1 Disease of nose	Factor 2 Incompetent lip seal	Problems with swallowing and chewing	Eating and drinking habits
Factor 1	1	0.320	0.235	−0.047
Factor 2		1	0.237	0.189
Factor 3			1	0.151
Factor 4				1

## 4 DISCUSSION

In our previous research, we asked the legal guardians of primary school children to fill out a questionnaire, and a factor analysis was performed to classify closely related questions into their respective factors (Saitoh et al., 2018). As a result, 26 of the 44 questions which might be linked to MBS were selected, and they were classified into seven factors as follows: ILS, diseases of the nose and throat, eating and drinking habits, bad breath, the problem with swallowing and chewing, condition of teeth and gums, and dry lips (Saitoh et al., 2018). In contrast, there was less extraction of the MBS‐related factors with only nine items for preschool children in this study, and the nine items were classified into four factors as follows: diseases of the nose, ILS, problems with swallowing and chewing, and eating and drinking habits. While the questionnaire for this study was based on a two‐point scale (yes or no), the questionnaire for the previous research was based on a four‐point scale (yes, maybe yes, maybe no, or no). Therefore, it is not possible to simply compare the results of both.　However, ILS, diseases of the nose, problems with swallowing and chewing, and eating and drinking habits were the common extracted factors for MBS in both studies. The results suggest that the initial stages of multiple diseases were present among preschool children. These diseases and problems might aggravate as the child grows.

ILS had a high correlation with problems in swallowing and chewing and eating and drinking habits and the highest correlation with diseases of the nose. Sabashi et al. (2011) investigated the relationship between nasal obstruction and lip‐closing force in adults and reported that the lip‐closing force in the nasal obstruction group was significantly less than for the normal group, and nasal airway resistance and lip‐closing force showed a negative correlation in the nasal obstruction group. Pacheco et al. (2015) investigated the association between mouth breathing and upper airway narrowing in childhood and reported that 32.5% of mouth breathers had severe hypertrophy of the palatine tonsils, 53.9% had atretic palate, and 35.9% had lip incompetence. Human infants are considered to be nasal breathers, and they can breathe through the mouth according to their developmental stage of eating and articulation function (Harding et al., 1995; van Someren & Stothers, 1983). The upper airway resistance due to nasal obstruction causes the transition to vicarious mouth breathing (Fitzpatrick et al., 2003; Trabalon & Schaal, 2012). It was thought that early diagnosis and treatment of the diseases of the nose is important for the achievement and maintenance of normal lip closing function, because of the strong correlation between ILS and diseases of the nose at 3–5 years of age.

Further, ILS and diseases of the nose were related to problems with swallowing and chewing. The inferior orbicularis oris muscle shows increased activity with jaw opening and consistent reciprocal cyclic activity with the posterior temporalis muscle in terms of temporal associations (Takada et al., 2018). Tomiyama et al. (2004) recorded the electromyographic (EMG) activity of the lower lip muscles and masseter during chewing and compared the differences between individuals with competent and incompetent lips. Subjects with incompetent lips showed higher EMG activities at rest and when the lips were in contact during chewing, compared to those with competent lips. Additionally, the duration of nonactive and total phases of the masseter when chewing with lip contact was shorter in the incompetent lip group than in the competent lip group. Their research suggested that ILS is a lip dysfunction and could affect masticatory function (Tomiyama et al., 2004). Macedo and Bianchini (2014) reported that the patients with orofacial myofunctional disorders showed mandibular movements with deviations and joint noises, amplitude reduction in lateral and protrusive movement, unilateral chewing, and excessive contraction of the orbicularis oris muscle during swallowing.

Meanwhile, Ikenaga et al. (2013) reported that mouth breathing induced by nasal obstruction decreased chewing activity and reduced the vertical effect on the posterior teeth. Lemos et al. (2009) concluded that the patients with nasal obstruction with rhinitis could not chew bilaterally with the lips closed or swallow water with the lips closed, tongue positioned on the palatine papilla, and without the inclusion of the periorbicular muscles. The orbicularis oris muscle and nasal breathing played an important role in chewing and swallowing and had an influence on not only the form but also the function of the maxillofacial region.

We concluded that the initial stages of MBS may be present among preschool children, and MBS could become chronic with growth. ILS and diseases of the nose cause poor development of oral functions, including mouth breathing and difficulty in chewing and swallowing. Thus, early intervention for ILS and diseases of the nose, in collaboration with a pediatrician, otolaryngologist, and dentist, might improve the patients' facial form and oral function. Not only ILS, but also malocclusion and sleep‐related breathing disorders develop gradually starting at an early age (Di Carlo et al., 2020; Luzzi et al., 2017). Early first dental visits are important for the early detection of these diseases (Calcagnile et al., 2019). Pediatric dentists have a vital role in disseminating information about the oral health of children to their parents and in earlier diagnosis of these disorders.

## 5 CONCLUSION

To clarify factors relevant to MBS in preschool children and to investigate the relationship of ILS to MBS, we divided the closely related questions about daily health conditions and lifestyle habits into groups. The results regarding the correlations between the newly revealed factors support the following conclusions:

1. The questions were classified into four categories. Factors 1–4 were defined as “diseases of the nose,” “ILS,” “problems with swallowing and chewing,” and “eating and drinking habits,” respectively.
2. Factor 2, ILS, was most strongly correlated with Factor 1, diseases of the nose.
3. Factor 3, problems with swallowing and chewing, showed a relatively strong correlation with Factors 1 and 2.
4. Therefore, the initial stage of MBS may be present in preschool children, which may be related to the above‐described four factors, especially ILS.

Our findings could contribute to the early diagnosis and management of MBS for children and improve their long‐term quality of life.

## AUTHOR CONTRIBUTIONS

All authors were involved in the conception, design, data acquisition, data analysis, interpretation, drafting of the manuscript, and its critical revision. All authors gave final approval of the version to be published.

## CONFLICT OF INTEREST

The authors declare no conflict of interest.

## Data Availability

The data sets analyzed during the present study are not publicly available due to ethical restrictions but are available from the corresponding author on reasonable request. All data analyzed during this study are included in this published article.

## References

[cre2661-bib-0001] Brodie, A. D. (1954). The fourth dimension in orthodontia. Angle Orthodontist, 24(1), 15–30.

[cre2661-bib-0002] Calcagnile, F. , Pietrunti, D. , Pranno, N. , Di Giorgio, G. , Ottolenghi, L. , & Vozza, I. (2019). Oral health knowledge in pre‐school children: A survey among parents in central Italy. Journal of Clinical and Experimental Dentistry, 11(4), e327–e333. 31110611 10.4317/jced.55378 PMC6522113

[cre2661-bib-0003] Campanha, S. M. , Fontes, M. J. , Camargos, P. A. , & Freire, L. M. (2010). The impact of speech therapy on asthma and allergic rhinitis control in mouth breathing children and adolescents. Jornal de Pediatria, 86(3), 202–208. 20449526 10.2223/JPED.1995

[cre2661-bib-0005] Choi, J. E. , Waddell, J. N. , Lyons, K. M. , & Kieser, J. A. (2016). Intraoral pH and temperature during sleep with and without mouth breathing. Journal of Oral Rehabilitation, 43(5), 356–363. 26666708 10.1111/joor.12372

[cre2661-bib-0006] Conti, P. B. , Sakano, E. , Ribeiro, M. A. , Schivinski, C. I. , & Ribeiro, J. D. (2011). Assessment of the body posture of mouth‐breathing children and adolescents. Jornal de Pediatria, 87(4), 357–363. 21769416 10.2223/JPED.2102

[cre2661-bib-0004] Di Carlo, G. , Zara, F. , Rocchetti, M. , Venturini, A. , Ortiz‐Ruiz, A. J. , Luzzi, V. , Cattaneo, P. M. , Polimeni, A. , & Vozza, I. (2020). Prevalence of sleep‐disordered breathing in children referring for first dental examination. a multicenter cross‐sectional study using pediatric sleep questionnaire. International Journal of Environmental Research and Public Health, 17(22), 8460. 33207543 10.3390/ijerph17228460 PMC7698058

[cre2661-bib-0007] Fitzpatrick, M. F. , McLean, H. , Urton, A. M. , Tan, A. , O'Donnell, D. , & Driver, H. S. (2003). Effect of nasal or oral breathing route on upper airway resistance during sleep. European Respiratory Journal, 22(5), 827–832. 14621092 10.1183/09031936.03.00047903

[cre2661-bib-0008] Harari, D. , Redlich, M. , Miri, S. , Hamud, T. , & Gross, M. (2010). The effect of mouth breathing versus nasal breathing on dentofacial and craniofacial development in orthodontic patients. Laryngoscope, 120(10), 2089–2093. 20824738 10.1002/lary.20991

[cre2661-bib-0009] Harding, R. , Jakubowska, A. E. , & McCrabb, G. J. (1995). Postnatal development of responses to airflow obstruction. Clinical and Experimental Pharmacology and Physiology, 22(8), 537–543. 7586710 10.1111/j.1440-1681.1995.tb02063.x

[cre2661-bib-0010] Hsu, H. Y. , & Yamaguchi, K. (2012). Decreased chewing activity during mouth breathing. Journal of Oral Rehabilitation, 39(8), 559–567. 22490018 10.1111/j.1365-2842.2012.02306.x

[cre2661-bib-0011] Ikenaga, N. , Yamaguchi, K. , & Daimon, S. (2013). Effect of mouth breathing on masticatory muscle activity during chewing food. Journal of Oral Rehabilitation, 40(6), 429–435. 23566154 10.1111/joor.12055

[cre2661-bib-0012] Inada, E. , Saitoh, I. , Kaihara, Y. , Murakami, D. , Nogami, Y. , Kubota, N. , Shirazawa, Y. , Ishitani, N. , Oku, T. , & Yamasaki, Y. (2021). Incompetent lip seal affects the form of facial soft tissue in preschool children. Cranio, 39(5), 405–411. 31469617 10.1080/08869634.2019.1656936

[cre2661-bib-0013] Jung, M. H. , Yang, W. S. , & Nahm, D. S. (2010). Maximum closing force of mentolabial muscles and type of malocclusion. Angle Orthodontist, 80(1), 72–79. 19852643 10.2319/020509-78.1 PMC8978727

[cre2661-bib-0014] Kotecha, B. (2011). The nose, snoring and obstructive sleep apnoea. Rhinology, 49(3), 259–263. 21858254 10.4193/Rhino10.165

[cre2661-bib-0015] Lemos, C. M. , Wilhelmsen, N. S. , Mion Ode, G. , & Mello Junior, J. F. (2009). Functional alterations of the stomatognathic system in patients with allergic rhinitis: Case‐control study. Brazilian Journal of Otorhinolaryngology, 75(2), 268–274. 19575115 10.1016/S1808-8694(15)30789-8 PMC9450752

[cre2661-bib-0016] Lofstrand‐Tidestrom, B. , Thilander, B. , Ahlqvist‐Rastad, J. , Jakobsson, O. , & Hultcrantz, E. (1999). Breathing obstruction in relation to craniofacial and dental arch morphology in 4‐year‐old children. European Journal of Orthodontics, 21(4), 323–332. 10502895 10.1093/ejo/21.4.323

[cre2661-bib-0017] Luzzi, V. , Ierardo, G. , Corridore, D. , Di Carlo, G. , Di Giorgio, G. , Leonardi, E. , Campus, G. G. , Vozza, I. , Polimeni, A. , & Bossù, M. (2017). Evaluation of the orthodontic treatment need in a paediatric sample from Southern Italy and its importance among paediatricians for improving oral health in pediatric dentistry. Journal of Clinical and Experimental Dentistry, 9(8), e995–e1001. 28936290 10.4317/jced.54005 PMC5601117

[cre2661-bib-0018] Macedo, P. F. , & Bianchini, E. M. (2014). Myofunctional orofacial examination: Comparative analysis in young adults with and without complaints. Codas, 26(6), 464–470. 25590908 10.1590/2317-1782/20142014015

[cre2661-bib-0019] Mizuno, R. , Yamada, K. , Murakami, M. , Kaede, K. , & Masuda, Y. (2014). Relationship between frontal craniofacial morphology and horizontal balance of lip‐closing forces during lip pursing. Journal of Oral Rehabilitation, 41(9), 659–666. 24889375 10.1111/joor.12190

[cre2661-bib-0020] Moyers, M. E. (1972). Handbook of orthodontics (3rd ed., pp. 730–743). Year Book Medical Publishers Inc.

[cre2661-bib-0021] Nagaiwa, M. , Gunjigake, K. , & Yamaguchi, K. (2016). The effect of mouth breathing on chewing efficiency. Angle Orthodontist, 86(2), 227–234. 26222411 10.2319/020115-80.1 PMC8603605

[cre2661-bib-0022] Nishiura, M. , Ono, T. , Yoshinaka, M. , Fujiwara, S. , Yoshinaka, M. , & Maeda, Y. (2015). Pressure production in oral vestibule during gum chewing. Journal of Oral Rehabilitation, 42(12), 900–905. 26147313 10.1111/joor.12328

[cre2661-bib-0023] Nogami, Y. , Saitoh, I. , Inada, E. , Murakami, D. , Iwase, Y. , Kubota, N. , Nakamura, Y. , Kimi, M. , Hayasaki, H. , Yamasaki, Y. , & Kaihara, Y. (2021). Prevalence of an incompetent lip seal during growth periods throughout Japan: A large‐scale, survey‐based, cross‐sectional study. Environmental Health and Preventive Medicine, 26(1), 11. 33478389 10.1186/s12199-021-00933-5 PMC7819306

[cre2661-bib-0024] Okuro, R. T. , Morcillo, A. M. , Ribeiro, M. A. , Sakano, E. , Conti, P. B. , & Ribeiro, J. D. (2011). Mouth breathing and forward head posture: Effects on respiratory biomechanics and exercise capacity in children. Jornal Brasileiro de Pneumologia: Publicaça̋o Oficial da Sociedade Brasileira de Pneumologia e Tisilogia, 37(4), 471–479. 21881737 10.1590/s1806-37132011000400009

[cre2661-bib-0025] Pacheco, M. C. , Fiorott, B. S. , Finck, N. S. , & Araujo, M. T. (2015). Craniofacial changes and symptoms of sleep‐disordered breathing in healthy children. Dental Press Journal of Orthodontics, 20(3), 80–87. 26154460 10.1590/2176-9451.20.3.080-087.oar PMC4520142

[cre2661-bib-0026] Sabashi, K. , Washino, K. , Saitoh, I. , Yamasaki, Y. , Kawabata, A. , Mukai, Y. , & Kitai, N. (2011). Nasal obstruction causes a decrease in lip‐closing force. Angle Orthodontist, 81(5), 750–753. 21446868 10.2319/103010-639.1 PMC8916196

[cre2661-bib-0027] Saitoh, I. , Inada, E. , Kaihara, Y. , Nogami, Y. , Murakami, D. , Kubota, N. , Sakurai, K. , Shirazawa, Y. , Sawami, T. , Goto, M. , Nosou, M. , Kozai, K. , Hayasaki, H. , & Yamasaki, Y. (2018). An exploratory study of the factors related to mouth breathing syndrome in primary school children. Archives of Oral Biology, 92, 57–61. 29753207 10.1016/j.archoralbio.2018.03.012

[cre2661-bib-0028] van Someren, V. , & Stothers, J. K. (1983). A critical dissection of obstructive apnea in the human infant. Pediatrics, 71(5), 721–725. 6403917

[cre2661-bib-0029] Takada, J. I. , Miyamoto, J. J. , Sato, C. , Dei, A. , & Moriyama, K. (2018). Comparison of EMG activity and blood flow during graded exertion in the orbicularis oris muscle of adult subjects with and without lip incompetence: A cross‐sectional survey. European Journal of Orthodontics, 40(3), 304–311. 29016842 10.1093/ejo/cjx061 PMC5972603

[cre2661-bib-0030] Tomiyama, N. , Ichida, T. , & Yamaguchi, K. (2004). Electromyographic activity of lower lip muscles when chewing with the lips in contact and apart. Angle Orthodontist, 74(1), 31–36. 15038488 10.1043/0003-3219(2004)074<0031:EAOLLM>2.0.CO;2

[cre2661-bib-0031] Trabalon, M. , & Schaal, B. (2012). It takes a mouth to eat and a nose to breathe: Abnormal oral respiration affects neonates' oral competence and systemic adaptation. International Journal of Pediatrics, 2012, 207605. 22811731 10.1155/2012/207605 PMC3397177

[cre2661-bib-0032] Weinstein, S. , Haack, D. C. , Morris, L. Y. , Snyder, B. B. , & Attaway, H. E. (1963). On an equilibrium theory of tooth position. Angle Orthodontist, 33(1), 1–26.

